# Evaluation of large language models under different training background in Chinese medical examination: a comparative study

**DOI:** 10.3389/frai.2024.1442975

**Published:** 2024-12-04

**Authors:** Siwen Zhang, Qi Chu, Yujun Li, Jialu Liu, Jiayi Wang, Chi Yan, Wenxi Liu, Yizhen Wang, Chengcheng Zhao, Xinyue Zhang, Yuwen Chen

**Affiliations:** ^1^School of Medical Device, Shenyang Pharmaceutical University, Shenyang, China; ^2^Department of Clinical Laboratory, Peking Union Medical College Hospital, Chinese Academy of Medical Sciences, Beijing, China; ^3^School of Pharmacy, Shenyang Pharmaceutical University, Shenyang, China; ^4^School of Pharmaceutical Engineering, Shenyang Pharmaceutical University, Shenyang, China; ^5^School of Business Administration, Shenyang Pharmaceutical University, Shenyang, China

**Keywords:** general large language model, medical large language model, CMLE, artificial intelligence, openAI

## Abstract

**Background:**

Recently, Large Language Models have shown impressive potential in medical services. However, the aforementioned research primarily discusses the performance of LLMs developed in English within English-speaking medical contexts, ignoring the LLMs developed under different linguistic environments with respect to their performance in the Chinese clinical medicine field.

**Objective:**

Through a comparative analysis of three LLMs developed under different training background, we firstly evaluate their potential as medical service tools in a Chinese language environment. Furthermore, we also point out the limitations in the application of Chinese medical practice.

**Method:**

Utilizing the APIs provided by three LLMs, we conducted an automated assessment of their performance in the 2023 CMLE. We not only examined the accuracy of three LLMs across various question, but also categorized the reasons for their errors. Furthermore, we performed repetitive experiments on selected questions to evaluate the stability of the outputs generated by the LLMs.

**Result:**

The accuracy of GPT-4, ERNIE Bot, and DISC-MedLLM in CMLE are 65.2, 61.7, and 25.3%. In error types, the knowledge errors of GPT-4 and ERNIE Bot account for 52.2 and 51.7%, while hallucinatory errors account for 36.4 and 52.6%. In the Chinese text generation experiment, the general LLMs demonstrated high natural language understanding ability and was able to generate clear and standardized Chinese texts. In repetitive experiments, the LLMs showed a certain output stability of 70%, but there were still cases of inconsistent output results.

**Conclusion:**

General LLMs, represented by GPT-4 and ERNIE Bot, demonstrate the capability to meet the standards of the CMLE. Despite being developed and trained in different linguistic contexts, they exhibit excellence in understanding Chinese natural language and Chinese clinical knowledge, highlighting their broad potential application in Chinese medical practice. However, these models still show deficiencies in mastering specialized knowledge, addressing ethical issues, and maintaining the outputs stability. Additionally, there is a tendency to avoid risk when providing medical advice.

## 1 Introduction

OpenAI has released ChatGPT (OpenAI, 2022), a natural language understanding tool based on the Large Language Models (LLMs) in November 2022, which quickly became globally recognized for its extensive knowledge and human-like communication abilities. Building on this, GPT-4 (OpenAI, 2023) was introduced in March 2023 with multi-modal inputs, enhancing its ability to handle complex tasks. Many experts believe GPT-4 may be a precursor to Artificial General Intelligence (AGI). The success of ChatGPT or GPT-4 is attributed to the LLMs with an immense parameters, which can be pre-trained on massive text data, thus mastering rich knowledge and possessing strong contextual understanding and text generation capabilities. As a result, it has been widely applied in numerous scenarios (Patel and Lam, 2023).

The LLMs has also shown the great potential in medical domain. Nori et al. (2023) conducted a comprehensive evaluation of GPT-4’s application in the medical domain and found that it scored 20 points above the passing score in the United States Medical Licensing Examination (USMLE), demonstrating its significant potential in medical education, assessment, and clinical practice. Butler et al. (2024) explored the application of ChatGPT in predicting and preventing ankle injuries, discovering that it could help doctors improve the diagnostic accuracy of foot and ankle diseases and customize personalized rehabilitation plans for patients, while tracking recovery progress in real-time. Additionally, dental monitoring software with LLMs (Fatani, 2023) has enhanced the daily collaboration between dental clinics and patients through smartphone applications, optimizing the coordination and outcome monitoring of the treatment process. Strunga et al. (2023) believe that artificial intelligence shows great potential in improving the quality of patient care and the outcomes of orthodontic treatment. By presenting the same neurosurgical questions to GPT-4 and other GPT models, GPT-4’s outstanding performance highlights its potential value in integrating patient care. Brin et al. (2023) assessment indicates that GPT-4’s accuracy in medical certification exams is significantly higher than ChatGPT. Grünebaum et al. (2023) shows that the performance of ChatGPT in monitoring and preventing preterm birth is unsatisfactory, revealing the incompleteness of the answers. Wu et al. (2023) indicates that although GPT-4 exhibits professional-level performance in identifying medical imaging modalities and anatomical knowledge, it still faces challenges in disease diagnosis and writing comprehensive medical reports.

Medical scenarios are too specialized for general LLMs, which are prone to generating hallucinations. Therefore, some researchers (Li et al., 2023; Touvron et al., 2023; Singhal et al., 2023) have developed domain-specific LLMs from general LLMs. These domain-specific LLMs undergo a fine-tuning process with domain datasets, enhancing their capability to handle specific tasks. For example, ChatDoctor (Li et al., 2023) is a medical LLM that has been fine-tuned on the LLaMA (Touvron et al., 2023), utilizing patient-physician dialogues as its training dataset. It aims to bridge the communication gap between patients and healthcare providers by understanding and generating medically relevant responses. Med-PaLM2 (Singhal et al., 2023) is developed by Google, which has shown exceptional promise for clinical applications, achieving high scores on multiple benchmarks.

The aforementioned research primarily tests the performance of LLMs developed in English within English-speaking medical contexts. However, their efficacy and applicability in medical practices under other languages, such as Chinese, have not yet been sufficiently studied and validated. In other words, there exists a gap concerning the comparative analysis of LLMs developed under different linguistic environments with respect to their performance in the Chinese clinical medicine field. Therefore, this study presents a novel evaluation of GPT-4 (a general LLM mainly developed in English), ERNIE Bot (a general LLM mainly developed in Chinese), and Disc-MedGPT (a specialized medical LLM), assessing their capabilities in understanding Chinese medical knowledge, clinical treatment protocols, legal and ethical. This study also delves into potential issues that LLMs may encounter in the application of Chinese medical practice and offers corresponding recommendations for improvement. This research is anticipated to not only foster the integration of LLMs in the domain of Chinese medicine but also to offer novel perspectives and methodologies for medical information processing and knowledge transfer within cross-linguistic and cross-cultural contexts.

## 2 Materials and methods

### 2.1 Data source and dataset construction

We have chosen the 2023 Chinese Medical Licensing Examination (CMLE) as our data source for evaluating Large Language Models (LLMs) and have selected 600 single-choice questions from the clinical physician section. These questions, along with their options and correct answers, have been compiled into a structured dataset for our research. For transparency, a sample of this dataset is presented in Table 1.

**Table 1:** Sample of the dataset.

No	Question	Option A	Option B	Option C	Option D	Option E	Correct answer	Unit
1	A 23-year-old woman found a smooth, movable lump in her right breast while taking a bath. She went to the hospital and was diagnosed with fibrocystic disease of the breast. The pathological feature of this condition is:	Proliferating glands are squeezed into slit-like shapes by fibers.	There is often bloody nipple discharge.	Painful solitary lump.	Fibrocystic disease of the breast is most common in the upper inner quadrant of the breast, round or oval in shape.	The most common malignant tumor of the female breast.	A	U1
2	In 2017, a city’s health bureau initiated an election for “advanced departments” and selected 30 “outstanding advanced departments.” In this positive environment, all hospitals are striving to be “advanced departments.” The psychological factor that promotes this is:	Public opinion can influence people through the environment.	Case analysis to persuade people with reason.	Technical proficiency is paramount.	Setting an example to inspire people.	Educating with knowledge to cultivate morality.	D	U1
3	The relationship between the age X (in years) and the average weight Y (in kg) of children aged 2–7 can be represented as *Y* = 7 + 2X. Which of the following is correct:	The average weight of a 0-year-old child is 7 kg.	The average weight of an 11-year-old child is 29 kg.	The average weight of a 5-year-old child is 17 kg.	A 6-year-old child has an average weight 7 kg more than a 5-year-old.	From 1 to 2 years old, the weight increases by 2 kg.	C	U1
4	The national selection of a certain range of areas for AIDS prevalence, through a unified method and approach to survey the general population and high-risk groups in the area, this statistical method belongs to:	Passive surveillance.	Mortality surveillance.	Symptom surveillance.	Sentinel surveillance.	Behavioral surveillance.	D	U1
5	A 25-year-old man injured his foot while mowing grass, which did not improve after ten days of herbal treatment. Physical examination: T38.0°C, P72 beats/min, R24 breaths/min, redness and swelling of the foot, and a crepitus under the skin of the dorsum of the foot, with indistinct boundaries from surrounding tissues. Pathology showed diffuse infiltration of neutrophils, the lesion might be:	Fibrinous inflammation.	Serous inflammation.	Abscess.	Gangrene.	Cellulitis.	E	U1

### 2.2 Selection of large language models

To explore the capabilities of various LLMs in the Chinese medical context across different linguistic environments, we have chosen two general LLMs: OpenAI’s GPT-4 and Baidu’s ERNIE Bot, as well as a medical LLM called DISC-MedLLM, as the objects of our study.

#### 2.2.1 GPT-4

GPT-4 represents the state-of-the-art in LLMs, with its training corpus primarily sourced from English materials. Consequently, it is considered an appropriate representative to assess the capabilities of Chinese medical question-answering in a cross-linguistic environment (OpenAI, 2023).

#### 2.2.2 Baidu’s ERNIE Bot

ERNIE Bot is the latest LLM introduced by Baidu and is one of the earliest LLMs developed in China. This model has been optimized for Chinese natural language understanding and generation tasks to better serve Chinese application scenarios. Therefore, we have selected it as a representative of Chinese LLMs (AI Studio, n.d.).

#### 2.2.3 DISC-MedLLM

DISC-MedLLM is a medical LLM designed for healthcare dialogue scenarios. It is derived from the Baichuan-13B-Base (OpenAI, 2023). Through high-quality supervised fine-tuning datasets, which include medical knowledge graphs, reconstructed real conversations, and human preference rephrasing, this model excels in both single-turn and multi-turn medical consultation scenarios, making it a representative of medical LLMs in our research.

It is worth noting that this model has a substantially smaller parameter count than the two aforementioned general LLMs. (Bao et al., 2023).

### 2.3 Experiment setup

#### 2.3.1 Accuracy experiment

We utilized the Python programming language to script calls to the official data interfaces, feeding the datasets into the three models. The outcomes were returned in JSON format and compiled into formatted documents. To ensure accuracy, the prompt instructed the models to assume the role of an experienced physician addressing complex medical inquiries. The models were asked to return the correct answer and its explanation, formatted as specified in the JSON structure. The scripts and results are available to the public for download.^1^

#### 2.3.2 Chinese text generation experiment

In CMLE Testing, an additional requirement was set for generating explanations for answers. Four medical students conducted further analysis of these explanations to ascertain the correctness of the model responses and to evaluate the Chinese text generation capabilities of each LLM.

#### 2.3.3 Repetition experiment

To assess the stability of LLMs in medical application scenarios, we randomly selected 30 medical questions and subjected each to seven rounds of repeated testing.

## 3 Result

### 3.1 Overview

#### 3.1.1 Overall accuracy

After rigorous testing, we fed the dataset to GPT-4, ERNIE Bot, and DISC-MedLLM, yielding overall accuracies of 65.2, 61.7, and 25.3%, respectively. It can be observed that both GPT-4 and ERNIE Bot, which are general LLMs, met the criteria for the CMLE. Moreover, their performance significantly exceeded that of the medical LLM, DISC-MedLLM. Notably, GPT-4 demonstrated the highest accuracy among the three LLMs, indicating no significant difference in Chinese language comprehension among LLMs developed across diverse linguistic contexts.

#### 3.1.2 Category analysis

**Figure 1:**
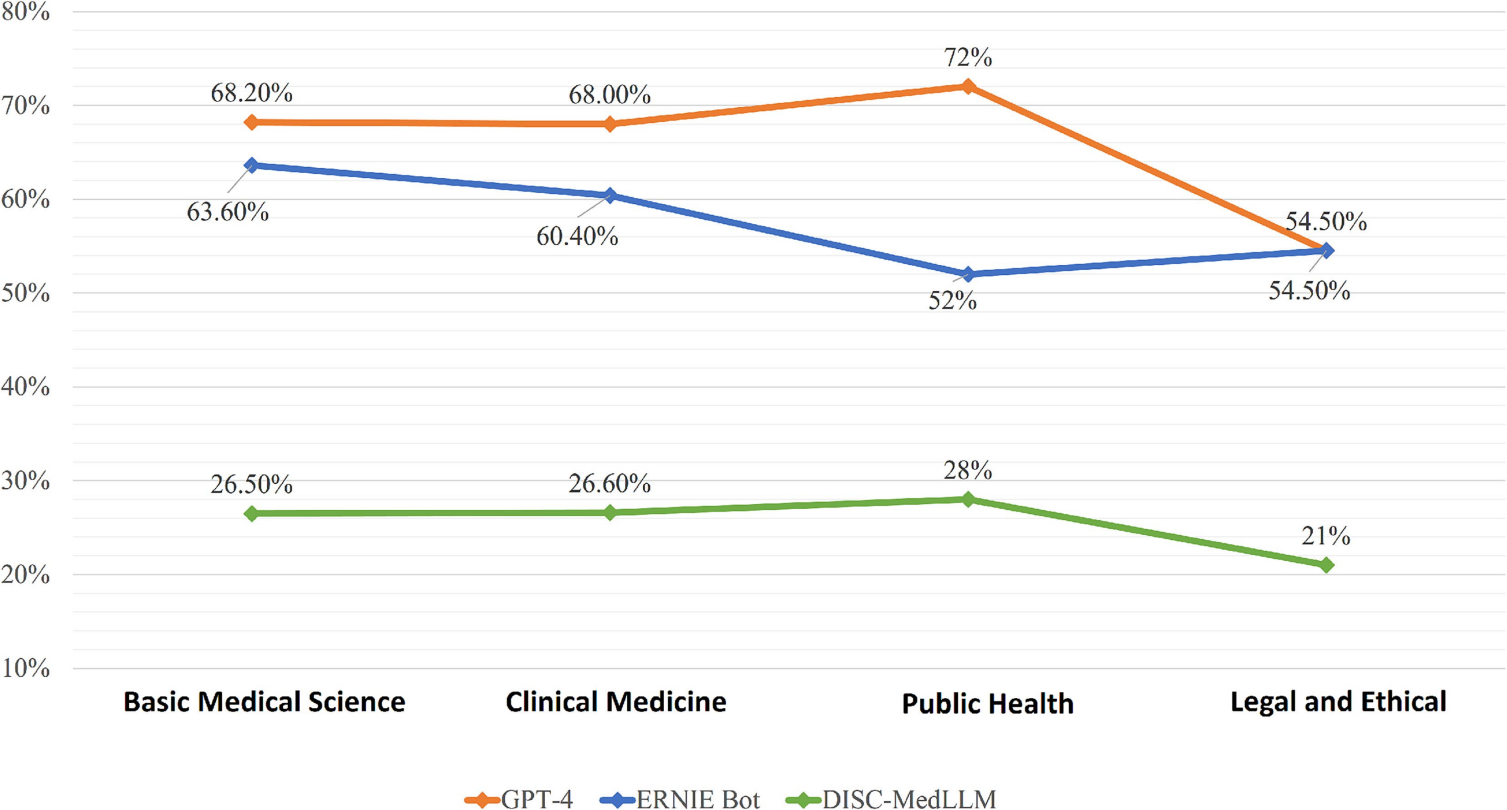
Accuracy of GPT-4, ERNIE Bot, DISC-MedLLM in four catagories.

**Figure 2:**
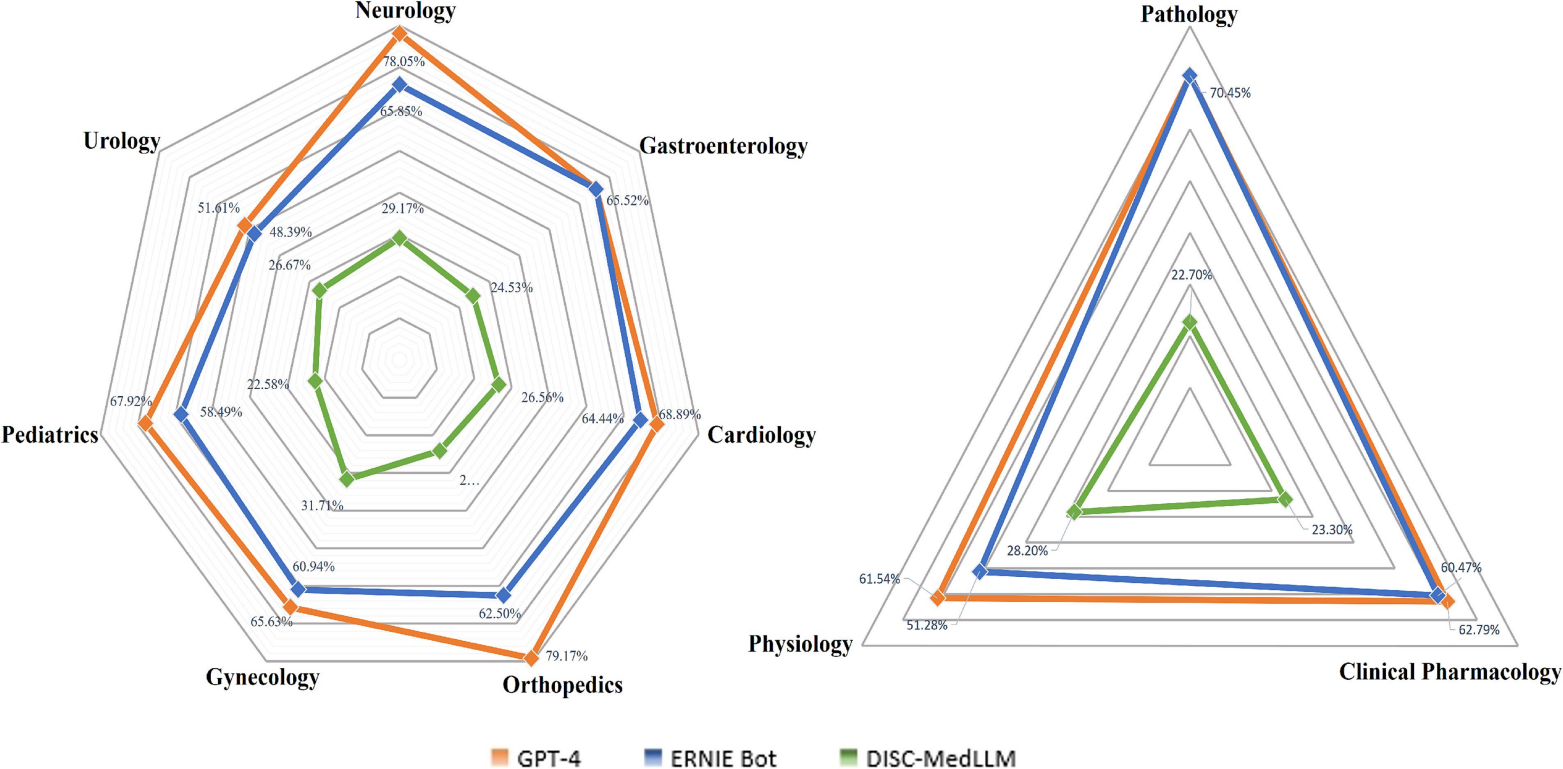
Comparison of clinical medicine and basic medicine science of LLMs.

The questions were categorized into Clinical Medicine, Basic Medical Sciences, Public Health, and Legal and Ethical, and analyzed in depth. As shown in Figure 1, GPT-4, ERNIE Bot, and DISC-MedLLM demonstrated descending average accuracies of 65.7, 57.6, and 25.6%. GPT-4 excelled in Public Health with an accuracy of 72%, while showing lower performance in Legal and Ethical at 54.5%. ERNIE Bot performed best in Basic Medical Sciences with an accuracy of 63.6% but was least accurate in Public Health at 52%. DISC-MedLLM showed uniformly lower accuracies across all categories, with its highest being 28% in Public Health and lowest at 21.2% in Legal and Ethical. We refined the Basic Medical Science and Clinical Medicine categorizes, with the specific data presented in Figure 2.

#### 3.1.3 Medical subject classification analysis

We categorized the questions into 14 medical subjects, including Pathology, Clinical Pharmacology, Physiology, Orthopedics, and others, as detailed in Table 2. It reveals that GPT-4 achieved the highest accuracy in 10 out of the 14 subject categories. Notably, ERNIE Bot surpassed GPT-4 in Legal Regulations with an accuracy of 60% compared to 45%. However, GPT-4’s performance in Ethical reached 69.2%, significantly surpassing ERNIE Bot’s 46.15%. DISC-MedLLM demonstrated its highest accuracy in Ethical at 38.5%.

**Table 2:** Accuracy by medical subjects.

Medical subject	GPT-4		ERNIE Bot		DISC-MedLLM	
	Correct amount	Accuracy	Correct amount	Accuracy	Correct amount	Accuracy
Pathology *n* = 44	31	70.45%	31	70.45%	10	22.73%
Clinical Pharmacology *n* = 43	27	62.79%	26	60.47%	10	23.26%
Physiology *n* = 39	24	61.54%	20	51.28%	11	28.21%
Orthopedics *n* = 24	19	79.17%	15	62.50%	7	29.17%
Pediatrics *n* = 53	36	67.92%	31	58.49%	13	24.53%
Gynecology *n* = 64	42	65.63%	39	60.94%	17	26.56%
Gastroenterology *n* = 58	38	65.52%	38	65.52%	14	24.14%
Neurology *n* = 41	32	78.05%	27	65.85%	13	31.71%
Urology *n* = 31	16	51.61%	15	48.39%	7	22.58%
Cardiology *n* = 45	31	68.89%	29	64.44%	12	26.67%
Health statistics *n* = 17	14	82.35%	11	64.71%	3	17.65%
Ethical *n* = 13	9	69.23%	6	46.15%	5	38.46%
Regulation *n* = 20	9	45.00%	12	60.00%	2	10.00%
Other *n* = 108	77	71.30%	63	58.33%	34	31.48%

#### 3.1.4 Case analysis

**Figure 3:**
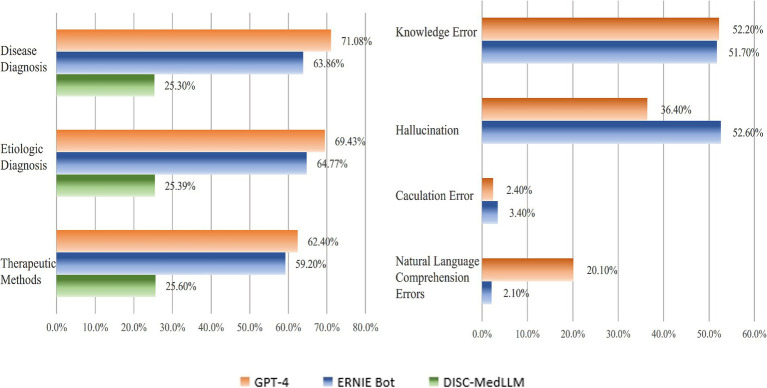
Accuracy of case and error questions.

We categorized questions which related to diagnostic scenarios, etiological determinations, and therapeutic approaches under the case analysis questions. These types of questions require the LLMs integrate patient information to diagnose specific diseases, identify causative factors, or directly propose treatment plans. Compared to other questions, case analysis questions demand a higher level of natural language understanding and medical diagnostic capability. Figure 3 provides a detailed presentation of the accuracy rates of the three LLMs in case analysis questions. GPT-4 attained an accuracy rate of 67.6%, ERNIE Bot achieved 62.8%, and DISC-MedLLM registered 25.4%. It is worth noting that, for all of the three LLMs, the accuracy rates for therapeutic approaches were the lowest within the case analysis category, with rates of 62.4, 59.2, and 25%, respectively.

#### 3.1.5 Errors analysis

Specific prompt was designed to elicit detailed explanations, which revealed that GPT-4 and ERNIE Bot understood the prompts well and could articulate relevant explanations. DISC-MedLLM, however, failed to grasp the prompt, failed to generate explanations. We categories the errors to knowledge errors (incomplete or inaccurate medical knowledge), hallucinations (logical coherence with incorrect content), natural language processing errors (misunderstandings of the question or prompt), and computational errors (incorrect computations). Figure 3 showed the result of errors analysis.

The knowledge errors in GPT-4 accounted for 52.2%, while ERNIE Bot’s for 51.7%. GPT-4 had a 36.4% rate of hallucinations, whereas ERNIE Bot’s was 52.6%. Natural language understanding errors were 20.1% for GPT-4 and just 2.1% for ERNIE Bot. Computational errors were 2.4 and 3.4%, respectively. These results highlight knowledge errors and hallucinations as the main issues. Moreover ERNIE Bot showed better comprehension skills in Chinese text compared to GPT-4. In computational tasks, GPT-4 outperformed ERNIE Bot with a 79.2% accuracy rate versus 66.7%.

### 3.2 Repetition experiment

The results indicated that in 70% of cases (21/30), the LLMs provided entirely consistent responses. However, inconsistencies were observed in the remaining 30% (9/30). These findings underscore the significant potential of LLMs in medical contexts, while also highlighting existing issues with output stability that necessitate further optimization.

### 3.3 GPT-4

GPT-4 achieved the best performance in our experiments (accuracy = 65.2%), benefitting from its vast parameter enables GPT-4 to process questions with minimal “hallucinations” (36.4%) and demonstrates its comprehensive knowledge, reaching the standard of the CMLE in 12 subjects. GPT-4 showed superior performance in computational and ethical questions with accuracy of 69.2 and 79.2%. In the experiment for generating explanations, GPT-4 was capable of producing clear and concise Chinese text, showcasing its exceptional ability in Chinese text generation.

### 3.4 ERNIE Bot

ERNIE Bot achieved an accuracy of 61.7%, meeting the standards of the CMLE. It excelled in legal regulations questions with an accuracy of 60%, surpassing both GPT-4 and DISC-MedLLM (45 and 10%). In the error analysis experiment, ERNIE Bot’s proportion of natural language understanding errors was a mere 2.1%. Moreover, in the Chinese text generation experiment, ERNIE Bot produced logically coherent and grammatically correct Chinese text. Results demonstrated that ERNIE Bot has been deeply optimized for the Chinese context, particularly excelling in Chinese legal regulations.

### 3.5 DISC-MedLLM

In this study, DISC-MedLLM achieved an accuracy of only 25.3%, failing to meet the pass standard of the CMLE. Additionally, in the Chinese text generation experiment, DISC-MedLLM demonstrated limited ability to comprehend prompts, failing to produce explanatory text.

## 4 Discussion

### 4.1 General LLMs vs. medical LLMs

GPT-4 and ERNIE Bot achieved accuracies of 65.2 and 61.7% respectively, meeting the qualification standards for Chinese clinical physicians, despite not being trained specifically for the medical domain. In contrast, DISC-MedLLM, a medical LLM, underperformed with an accuracy of 25.3%. This may be due to factors such as its smaller parameter, training data, and task adaptability. DISC-MedLLM, utilizing real doctor-patient dialogues to build a fine-tuning dataset, focuses more on performance in medical consultation scenarios rather than standardized examination settings (Bao et al., 2023). This distinction highlights the significant advantage of general LLMs in generalization over specialized models.

### 4.2 About error type

Through a comprehensive analysis of error types in general LLMs, we found that knowledge errors constitute a significant proportion of all errors. Specifically, the knowledge error rate for GPT-4 is 52.2% (109/209), while for ERNIE Bot, it is 51.7% (108/233). This highlights the limitations of current LLMs in handling knowledge-intensive queries despite their excellent performance across many domains. These limitations partly stem from the models’ training cut-off dates, as they cannot access real-time updates or post-training knowledge developments. Retrieval-Augmented Generation (RAG) is a crucial tool, which provides LLMs with an external knowledge source, enabling them to retrieve from a specified knowledge base and combine this information to generate high-quality responses (Lewis et al., 2020).

### 4.3 About risk mitigation

When addressing medical questions, LLMs tend to prioritize safety, and avoid high-risk medical suggestions or treatments with severe side effects. This cautious approach reflects the LLMs’ high level of prudence in dealing with health-related issues. To enhance the performance in medical practice, developers should further refine the training data of LLMs to better balance safety and accuracy when addressing sensitive and potentially high-risk medical issues. The process of building the database should involve professional medical experts who can incorporate complex medical cases and high-risk medical scenarios, allowing LLMs to learn a wider range of pathological conditions and treatment plans for high-risk situations. By constructing a training dataset that includes clinical notes, medical imaging, and doctor-patient dialogues, the model’s comprehensive understanding and processing capabilities of medical information can be strengthened. Our research shows that although LLMs cannot yet conduct medical diagnostics and treatments independently of healthcare professionals, they have substantial potential to support both medical staff and patients.

### 4.4 About ethical problem

Overall, the performance of LLMs on ethical issues needs improvement. This phenomenon indicates they lack deep understanding in complex human emotions and social norms. Additionally, ethical issues are often closely tied to individual values and cultural backgrounds. While LLMs are trained on extensive data, they may fail to accurately reflect or respect the ethical concepts of specific communities when faced with global diversity and cultural differences (Çiftci et al., 2024). The shortcomings in handling ethical matters could become a significant barrier for the implementation of LLMs in medical practice, Consequently, future investigations should focus on integrating a substantial body of ethical and legal case studies reflective of the models’ practical applications into their training datasets. Such an approach is anticipated to bolster the models’ capacity to comprehend intricate human emotions and adhere to societal norms effectively.

### 4.5 Output stability

In repeated experiments, Large Language Models (LLMs) provided consistent responses in 70% of cases, with variability observed in the remaining 30%. These findings suggest that further optimization in data and algorithms is necessary to enhance the reliability and stability of outputs when dealing with complex medical information. Enhancing the training datasets with high-quality medical data, ensuring diversity and comprehensiveness, and incorporating medical expertise to augment the model’s comprehension are effective strategies to improve the performance of LLMs on medical tasks. Furthermore, developers should focus on continuously improving the model’s performance and adaptability through parameter tuning and data augmentation techniques.

## 5 Conclusion

We firstly explore the performance of LLMs developed under different linguistic environments in the field of Chinese clinical medicine. The results indicated that the general LLMs such as GPT-4 and ERNIE Bot exhibit excellence in Chinese natural language and Chinese clinical knowledge, highlighting their broad potential application in Chinese medical practice. However, these LLMs still show deficiencies in mastering specialized knowledge, addressing ethical issues, and maintaining the outputs stability. Additionally, there is a tendency to avoid risk when providing medical advice.

Although, at this stage, LLMs cannot perform medical diagnostics and treatments without the involvement of medical professionals, we believe that with advancing technology, LLMs will ultimately play a pivotal role in the medical field, offering vital support to both medical staff and patients.

## Data Availability

The datasets presented in this study can be found in online repositories. The names of the repository/repositories and accession number(s) can be found in the article/supplementary material.

## Footnotes

^1^https://github.com/WuliZhuang/Evaluation-of-LLMs-Under-Different-Background
